# Study on the Carrier Transport Process in Deep Ultraviolet Light-Emitting Diodes with Al-Content-Varied AlGaN Composite Last Quantum Barrier

**DOI:** 10.3390/mi15121502

**Published:** 2024-12-16

**Authors:** Wei Liu, Yujia Liu, Junhua Gao, Zeyu Liu, Bohan Shi, Linyuan Zhang, Xinnan Zhao, Runzhi Wang

**Affiliations:** 1 School of Microelectronics, Northwestern Polytechnical University, Xi’an 710129, China; liuyujia000809@mail.nwpu.edu.cn (Y.L.); 2021264377@mail.nwpu.edu.cn (Z.L.); bohanshi@mail.nwpu.edu.cn (B.S.); 2023303024@mail.nwpu.edu.cn (L.Z.); zhaoxinnan@mail.nwpu.edu.cn (X.Z.); 2023264508@mail.nwpu.edu.cn (R.W.); 2 Chongqing Engineering Research Center of New Energy Storage Devices and Applications, Chongqing University of Arts and Sciences, Chongqing 402160, China

**Keywords:** ultraviolet light-emitting diodes, AlGaN quantum wells, electron blocking layer, quantum barriers, carrier transport

## Abstract

Serious electron leakage and poor hole injection efficiency are still challenges for deep ultraviolet AlGaN-based light-emitting diodes with a traditional structure in achieving high performance. Currently, the majority of research works concentrate on optimizing the structures of the electron blocking layer (EBL) and last quantum barrier (LQB) separately, rather than considering them as an integrated structure. Therefore, in this study, an Al-content-varied AlGaN composite last quantum barrier (CLQB) layer is proposed to replace the traditional EBL and LQB layers. It is found that when the Al content in the CLQB decreases from 70% to 60% along the growth direction, the sample’s luminescence efficiency is improved, which can be ascribed to the higher carrier concentration in the multiple quantum well active region caused by suppressed electron leakage and enhanced hole injection. Additionally, in the CLQB structure, the carrier loss at the EBL/LQB hetero-interface, which is inevitable in the traditional structure, can be avoided. However, if the Al content in the CLQB changes in an opposite way, i.e., increasing from 60% to 70%, the device optoelectronic performance deteriorates, since the electron leakage is enhanced and the hole injection is suppressed.

## 1. Introduction

The ternary compound aluminum gallium nitride (AlGaN) alloy is an important direct and wide bandgap semiconductor material. By adjusting the Al content, the bandgap can be tuned from 6.2 eV to 3.4 eV, covering the light wavelength range from 200–365 nm. In particular, due to its wide bandgap, the AlGaN material with a high Al content exhibits great potential in fabricating deep-ultraviolet light-emitting diodes (DUV LEDs), which can be used for disinfection and sterilization [1]. Compared to the traditional mercury-based sterilamp, the all-solid DUV LEDs based on AlGaN/AlGaN multiple quantum well (MQW) structures have the advantages of non-toxicity, environmental friendliness, high energy efficiency, long lifespan, and compact size, which have promising application prospects in food disinfection, air and water purification, and UV curing [2,3,4,5,6,7]. However, as the Al content increases, the luminescence efficiency of DUV LED decays remarkably [8]. So far, the external quantum efficiency of most AlGaN-based DUV LEDs with emission wavelengths less than 280 nm is lower than 20% [9].

There are several factors limiting the performance improvement of AlGaN-based DUV LEDs, mainly including the poor crystal quality of high-Al-content AlGaN material [10,11], the strong polarization effect in AlGaN QWs, and the serious leakage of electrons [12]. Among these factors, the electron overflow is a critical issue. In DUV LEDs, the Al content in AlGaN QW is much higher and even closer to that in AlGaN or AlN barriers to achieving DUV light emission [13]. Consequently, the potential well depth of AlGaN QW is shallow, which weakens its ability to capture and confine carriers, especially for electrons, and finally leads to severe electron overflow [14]. This results in a large amount of non-radiative loss of injected carriers, ultimately causing a significant reduction in luminescence efficiency. Typically, to solve this problem, a p-type electron blocking layer (EBL) with a high Al content is used to suppress electron overflow, thereby reducing electron leakage from the MQW active region to the p-side region.

However, the high-Al-content p-EBL also leads to an increase in the potential barrier height for hole injection into the active region. As a consequence, the p-EBL diminishes the efficiency of the hole injection [15]. In addition, the strong polarization effect between the EBL and the last quantum barrier (LQB) layer can cause a downward bend of the energy band, resulting in a charge accumulation at the EBL/LQB hetero-interface and hence hindering hole injection further [16,17]. Thus, several alternative designs for EBL structures were proposed, including a p-type Al_0.6_Ga_0.4_N/Al_0.5_Ga_0.5_N/p-Al_0.6_Ga_0.4_N EBL [18], an EBL with a gradually decreasing Al content [19], an EBL incorporating slope-shaped content [20], a trapezoidal EBL [21], and a quaternary AlInGaN EBL structure [22]. On the other hand, some novel structures for LQBs have also been developed, such as a compositional-graded quaternary LQB [23] and a Mg δ-doped last barrier (MDDLB) [24]. Additionally, to improve the hole injection efficiency and enhance the carrier confinement in the active region together, the interlayer structures, inserted between traditional separated LQB and EBL layers, have also been studied [25,26]. In recent years, further attempts have been made to enhance the p-region to effectively block electron entry while facilitating the efficient transport of holes into the MQWs. For instance, a single quaternary graded layer was proposed to replace conventional LQB, EBL, and p-AlGaN layers [27], and multi-gradient EBL and triangular LQB structures were verified to be able to significantly improve IQE and LOP [28], and electroluminescence (EL) measurements and ray-tracing simulations were developed to investigate the performance of LED with a p-SPSL structure [29].

However, so far, the majority of research works concentrate on optimizing the structures of the EBL and LQB separately, rather than considering them as an integrated structure. Therefore, in this paper, the traditional EBL and LQB are replaced with a novel integrated composite last quantum barrier (CLQB) structure, where the Al content varies continuously and linearly, to eliminate the lattice mismatch originated at the traditional EBL/LQB hetero-interface. Compared with the traditional EBL/LQB structures, the influence of CLQB structures with different distributions of Al content on the performance of AlGaN-based DUV LEDs is investigated in detail later.

## 2. Simulation and Model

**Figure 1:**
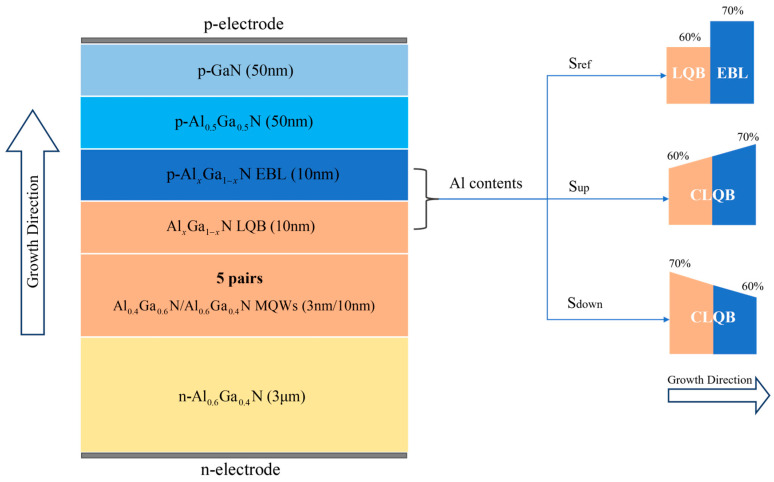
Schematic diagrams of samples S_ref_, S_up_, and S_down_.

The epitaxial structure of our DUV LEDs with lateral dimensions of 800 μm × 800 μm is composed of several critical components: a 3 μm thick n-type Al_0.6_Ga_0.4_N layer with doping concentration of 5 × 10^18^ cm^−3^, five pairs of MQW active regions consisting of 3 nm thick Al_0.4_Ga_0.6_N QW layers alternated with 10 nm thick Al_0.6_Ga_0.4_N quantum barrier (QB) layers [30], a 10 nm thick p-Al_0.7_Ga_0.3_N EBL with doping concentration of 1 × 10^19^ cm^−3^, a 50 nm thick p-Al_0.5_Ga_0.5_N layer with doping concentration of 2 × 10^19^ cm^−3^, and finally a 50 nm thick p-GaN Ohmic contact layer with doping concentration of 5 × 10^19^ cm^−3^. Since the size of our LEDs is much larger than the small-size LEDs such as micro-LEDs or mini-LEDs the surface nonradiative recombination process is ignored in our study. In the reference sample S_ref_, the traditional separated EBL and LQB structures are used, where the thickness of both EBL and LQB layers is 10 nm. The Al contents of EBL and LQB are 70% and 60%, respectively. In contrast, for the other two samples, labeled as S_up_ and S_down_, the CLQB structures with different varied Al contents are employed as a substitute for the traditional EBL and LQB layers. In sample S_up_, the Al content in CLQB linearly increases from 60% to 70% along the growth direction, while in sample S_down_, the Al content linearly decreases from 70% to 60% along the growth direction. The thickness of CLQB layers in samples S_up_ and S_down_ is equal to the total thickness of EBL and LQB layers in sample S_ref_, i.e., 20 nm. Additionally, in both CLQB layers, only the 10 nm region close to the p-Al_0.5_Ga_0.5_N layer is p-type-doped with a doping concentration of 1 × 10^19^ cm^−3^, to match the thickness and doping concentration of EBL in sample S_ref_. In short, the total Al content and thickness of the CLQB layers in S_up_ and S_down_ are equal to the sum of Al content and thickness of the EBL and LQB in sample S_ref_, respectively. However, the variation directions of Al content in CLQB layers are opposite for samples S_up_ and S_down_. The structural parameters of the rest regions are precisely the same for all samples. Figure 1 illustrates the schematic diagrams of the epitaxial structure of DUV LEDs studied in this work, as well as the different distributions of Al content in the EBL, LQB, and CLQB layers.

The optoelectronic properties of DUV LEDs are investigated numerically using Silvaco software(Silvaco 2021 TCAD), where the Schrödinger–Poisson and current continuity equations are solved self-consistently for the energy band profile and carrier distribution based on the carrier drift-diffusion model [31]. The radiative recombination and spectral luminescence are also calculated by solving the Schrödinger–Poisson equation based on a 3-band k·p model for Wurtzite-structure semiconductors [32]. During the simulation, the general radiative recombination rate, Auger recombination coefficient, and Shockley–Read–Hall (SRH) lifetime are set to be 1.1 × 10^−8^ cm^3^, 1 × 10^−34^ cm^6^/s, and 1 ns, respectively [33]. One should keep in mind that in the actual experiments, the lattice mismatch at hetero-interfaces, such as CLQB/QW interface, may cause lattice relaxation, generating crystal defects. However, if the influence of material quality is considered, the analysis and discussion may be much more complicated. In fact, our main purpose is to study the influence of CLQB structure itself on the transport properties of electrons and holes, rather than the actual crystal quality of AlGaN alloys. Therefore, for simplification, the crystal quality is set to be the same for all simulated DUV LEDs by setting an identical SRH lifetime for all samples. Considering that in the actual MQW structure, the polarization charges at the MQW hetero-interfaces may decrease, which may be induced by the lattice relaxation, the polarization coefficient during simulation is set to be 0.4 [34]. It is known that small-size electrodes can lead to current crowding effects, which may cause non-uniform distribution of radiative recombination. Therefore, in our simulations, for simplification, to achieve a uniform distribution of radiative recombination across the whole MQW active region, the vertical electrical injection structure is utilized in our samples, where the p-type and n-type electrodes are uniformly covered at the entire surfaces of the top of p-AlGaN and the bottom of n-AlGaN layers, respectively. In the simulation, the electrode is defined as a current boundary condition.

## 3. Results and Analysis

**Figure 2:**
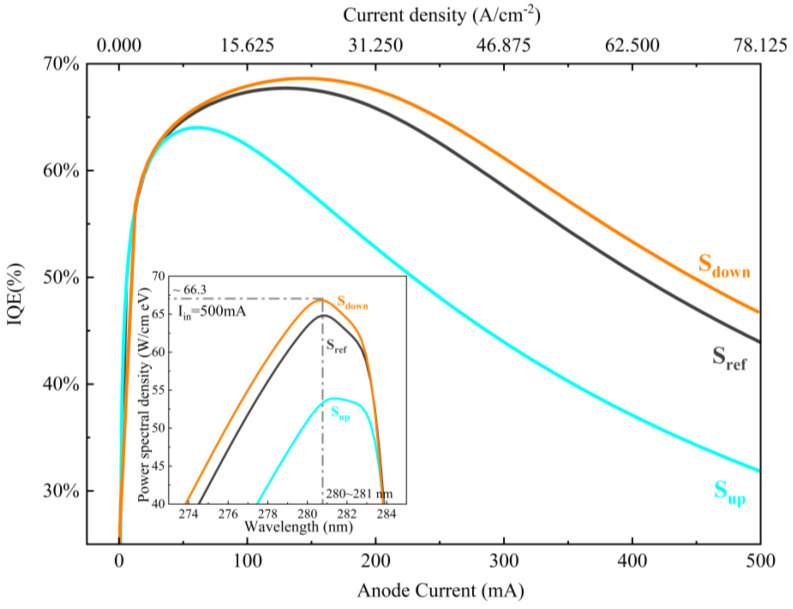
The IQE as a function of injection current for all samples. The inset shows the EL spectra of all of the samples at the injection current of 500 mA.

The variations of internal quantum efficiency (IQE) as functions of the forward injection current for samples S_ref_, S_up_, and S_down_ are presented in Figure 2. It is observed that the IQE values of all of the samples exhibit a significant reduction as the injection current increases, indicating a substantial efficiency droop effect. Among them, the values of IQE of sample S_down_ are the highest at all currents, i.e., they are 68.6% at the peak and 47.8% at the injection current of 500 mA. On the other hand, sample S_up_ shows the lowest IQE at all currents as well as the most severe efficiency droop. The EL spectra for all of the samples are displayed in the inset in Figure 2. The EL peak wavelengths for all of the samples are approximately 280 nm, indicating that the DUV light emission is achieved. It is seen that the wavelengths at the peak power spectral density of all of the samples are identical, which is reasonable and can be attributed to the same structural parameters of the MQW active region for all of the samples, such as Al content and QW thickness. However, the peak power spectral density decreases from samples S_down_ and S_ref_ to S_up_, which is consistent with the comparison result of their IQE values in Figure 2.

**Figure 3:**
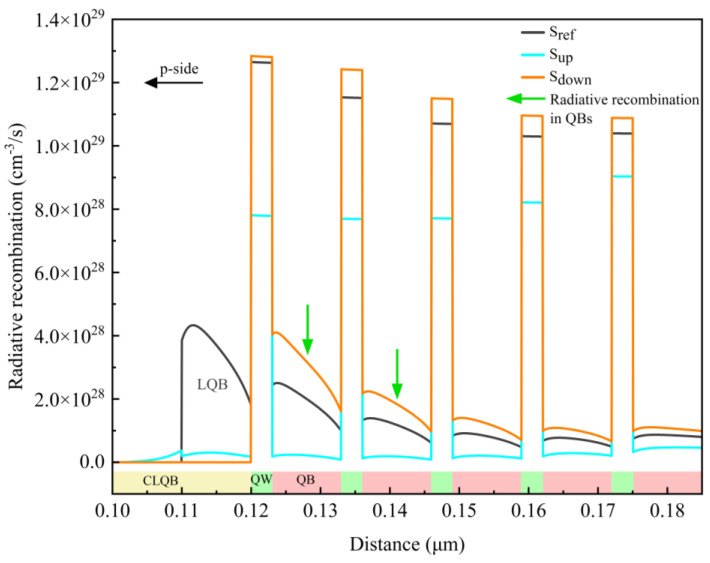
Distribution of radiative recombination rates in MQW active region for all samples. Green arrows indicate radiative recombination in quantum barriers.

To further explore the reason responsible for the differences in power spectral density in Figure 2, the radiative recombination rates across the entire MQW active region at the injection current of 500 mA are extracted and shown in Figure 3. It is obvious that, in sample S_down_, the radiative recombination rates in all five QWs are the highest, while those of sample S_up_ are significantly the lowest. It means that the comparison results of IQE and EL peak intensities presented in Figure 2 can be attributed to the different distribution of the radiative recombination rates in each individual sample. In addition, it is noteworthy that the radiative recombination rates in the QB layers of samples S_down_ and S_ref_ are significantly high, compared to sample S_up_, implying that there is a remarkable radiative recombination process in the QB layers in samples S_down_ and S_ref_. And especially for samples S_down_ and S_ref_, in the two QBs closest to the LQB or CLQB layer, the radiative recombination rates are noticeably enhanced, as indicated by the green arrows in Figure 3. Moreover, a significant enhancement of radiative recombination is observed within the LQB layer of sample S_ref_, which is not evident in the other two samples.

**Figure 4:**
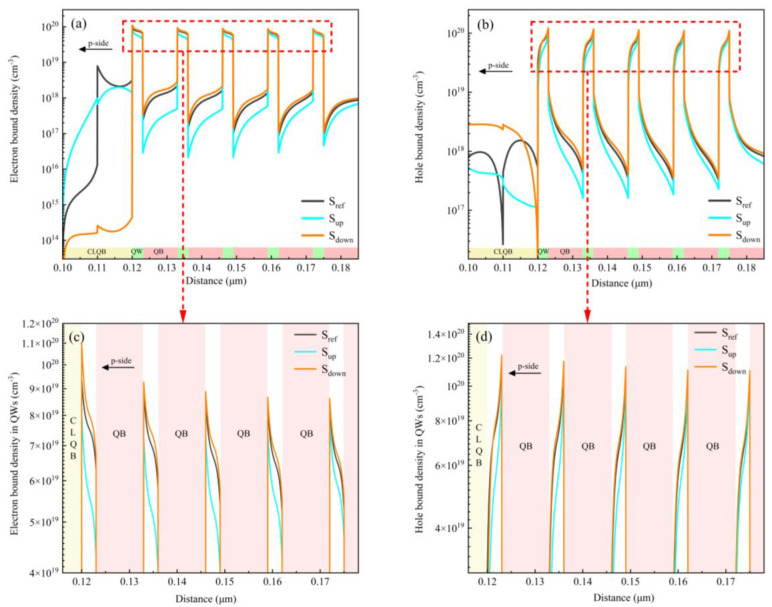
Distributions of (**a**) electrons and (**b**) holes in the entire MQW active region for all samples, as well as locally magnified views for (**c**) electron and (**d**) hole concentration in MQWs.

Figure 4 shows the distribution of carrier concentrations in the whole active region of all of the samples at the injection current of 500 mA. Logarithmic coordinates are used to clearly illustrate the variation of carrier concentrations in both QWs and QBs. Figure 4a,b present the distributions of electron and hole concentrations in the entire MQW region, respectively. For clearer observation, the distributions of electrons and holes in each QW are magnified and depicted in Figure 4c,d, respectively. It is seen that, in general, in the QWs, the hole and electron concentrations are the largest in sample S_down_, and they are the smallest in sample S_up_. It is well known that the radiative recombination process can be enhanced by increasing the carrier concentration. Therefore, since the electron and hole concentrations in the AlGaN QWs of sample S_down_ are the largest, the radiative recombination rate of sample S_down_ is the highest, which is consistent with the results in Figure 3.

Additionally, in Figure 4a,b, it is evident that the carriers exist not only in the QWs, but also in the QB layers. This is because in the DUV LEDs, the Al content in the AlGaN QW is relatively higher to achieve DUV light emission, which results in a shallower potential well due to the wider band gap of higher-Al-content AlGaN materials. As a consequence, the ability of AlGaN QWs to confine carriers is weakened. Therefore, at high injection currents, a portion of electrons and holes are not captured by the QWs and can be redistributed throughout the entire MQW active region as free carriers, especially in the AlGaN QBs. The high carrier concentrations in the QBs promote the occurrence of the radiative recombination process. According to Figure 4a,b, the electron and hole concentrations are the highest in the QBs of sample S_down_, leading to the largest radiative recombination rates in QBs in sample S_down_. On the contrary, for sample S_up_, where the carrier concentration in the QBs is much smaller, the radiative recombination process can be negligible, which is corroborated by the data of radiative recombination rates in QBs shown in Figure 3. Generally speaking, compared to samples S_ref_ and S_down_, the total carrier concentration in the MQW active region (including QWs and QBs) is much smaller for sample S_up_, which may be attributed to the weak electron confinement capability of the CLQB layer in the sample S_up_ and will be discussed later in detail.

For the comparison of the LQB and CLQB layers, it is observed that the peak concentrations of electrons and holes in the LQB of sample S_ref_ are approximately 10^19^ and 10^18^ cm^−3^, respectively. In contrast, they are about 10^18^ and 10^17^ cm^−3^ in the CLQB layer of sample S_up_, which is roughly ten times lower than those in sample S_ref_. Additionally, the electron concentration in the entire CLQB layer of sample S_down_ is less than 10^15^ cm^−3^. Therefore, since the carrier concentrations are very low in the CLQB, the radiative recombination process in the CLQB layer is suppressed significantly and can even be neglected, which is consistent with the recombination rate values in the CLQB in Figure 3. On the other hand, it is also observed that there is a significant accumulation of electrons and holes at the EBL/LQB hetero-interface of sample S_ref_. As a result, the radiative recombination process in its LQB layer is enhanced, leading to a higher radiative recombination rate in the LQB of sample S_ref_, as shown in Figure 3. In short, the significant carrier recombination loss in traditional EBL/LQB structures can be suppressed in our newly designed CLQB structure.

**Figure 5:**
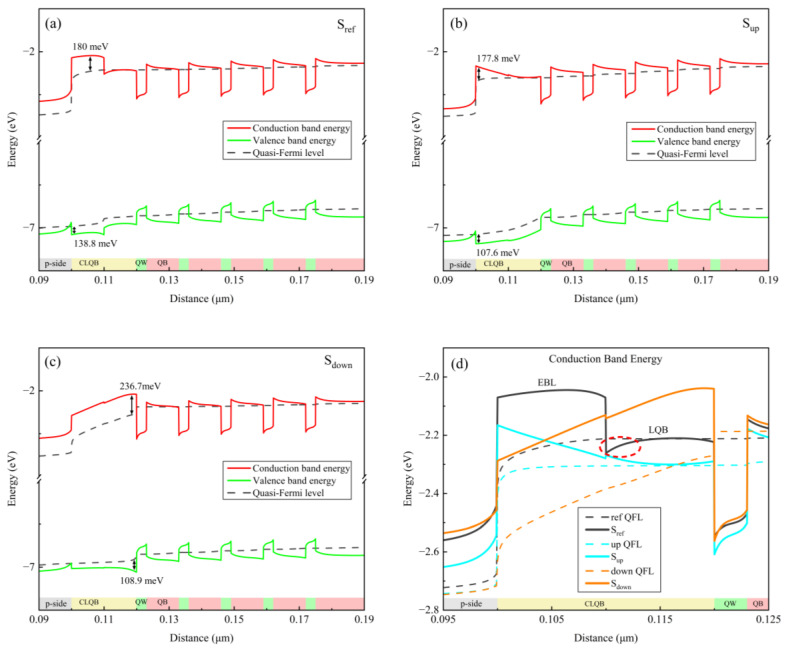
Energy band diagrams in the MQW active region of samples (**a**) S_ref_, (**b**) S_up_, (**c**) S_down_, and (**d**); comparison of conduction bands in the regions of EBL/LQB and CLQB for all samples at injection current of 500 mA. Dotted lines are electron quasi-Fermi levels, which are indicated by black, blue and orange, respectively. Red circle in (**d**) indicates downward bending of conduction band near interface between EBL and LQB.

In order to further investigate the variations of concentration distributions of electrons and holes, the energy band diagrams of all three samples were numerically extracted at an injection current of 500 mA, as depicted in Figure 5. Particularly, Figure 5d provides a detailed comparison of the conduction bands near the EBL/LQB or CLQB regions for all samples.

To evaluate the confinement ability of band structures on electrons and holes, we define the effective potential barrier height of electrons (or holes) as the distance between the highest point of the conduction band (or the lowest point of the valence band) and quasi-Fermi level of electrons (or holes) at the same position in the EBL for sample S_ref_ or in the CLQB for samples S_up_ and S_down_ [18]. It can be calculated that for samples S_ref_, S_up_, and S_down_, the effective barrier heights of electrons are 180, 177.8, and 236.7 meV, and those of holes are 138.3, 107.6, and 108.9 meV, respectively, which are all marked at the corresponding positions in Figure 5a, Figure 5b and Figure 5c, respectively.

For sample S_ref_, due to the difference in the Al content between the EBL and LQB layers, a strong piezoelectric polarization electric field is generated at the EBL/LQB hetero-interface, which causes the conduction band to bend downward in the LQB layer near the EBL/LQB interface [35,36]. Consequently, under high injection currents, a large number of electrons can readily fly over the LQB and then are blocked by the EBL, resulting in an accumulation of electrons at the EBL/LQB hetero-interface, as shown in Figure 4a. In fact, the electrons accumulated at the EBL/LQB interface can be regarded as a kind of leakage of electrons from MQWs, as it also leads to the reduction in the electron concentration in the MQW active region. Furthermore, plenty of injected holes are recombined with the accumulated electrons at the EBL/LQB interface before entering into the MQWs, leading to an enhanced radiative recombination process in the LQB layer of sample S_ref_, as illustrated in Figure 3. On the other hand, the effective potential barrier height of the holes is 138.3 meV for sample S_ref_, which is the highest potential barrier for the holes among all three samples. Due to the impact of the above several factors, i.e., the accumulated electrons at the EBL/LQB interface, the severe recombination loss of holes in the LQB, and the highest hole barrier in the EBL, both the electron and hole concentrations in the whole MQW active region are decreased. Consequently, the radiative recombination process in the MQWs is suppressed, and correspondingly, the recombination rates and carrier concentration are relatively smaller, as shown in Figure 3 and Figure 4.

For sample S_up_, the Al content in the CLQB layer increases linearly along the growth direction, forming a triangular potential barrier structure along the direction of electron injection, as seen in Figure 5b,d. Similar to the situation in sample S_ref_’s LQB, many electrons escaping from the MQWs can enter into the CLQB layer via the thermal emission process, since the difference in the Al content between the CLQB and the last QW is not very large, i.e., only 20%. On the other hand, within a triangular barrier, the effective potential barrier width is non-uniform; i.e., the closer to the top of triangular barrier, the thinner the effective barrier width is. It is well known from the basic quantum mechanics theory that the quantum tunneling effect can be facilitated in a thinner potential barrier [37,38]. Therefore, in sample S_up_, most electrons entering the CLQB may penetrate the upper part of the triangular CLQB barrier via quantum tunneling and then enter the p-type region, resulting in a serious electron leakage and, accordingly, a significant reduction in the carrier concentration and radiative recombination rates in the MQWs, as depicted in Figure 3 and Figure 4.

For sample S_down_, the Al content in the CLQB layer decreases linearly from 70% to 60% along the growth direction, forming an inverted triangular potential barrier structure along the direction of electron injection, as depicted in Figure 5d. Since the difference in the Al content between the CLQB and the last QW is as large as 30%, a high potential barrier is formed at the CLQB/QW hetero-interface, which effectively prevents electrons from entering the CLQB layer. Consequently, more electrons are confined within the MQW active region, resulting in an extremely low electron concentration in the CLQB, as shown in Figure 4a. On the other hand, compared to S_ref_, in sample S_down_, the inverted triangular CLQB barrier at the valance band can reduce the effective hole barrier height by approximately 30 meV. Moreover, the inverted triangular potential barrier structure at the valance band is also able to enhance the quantum tunneling process of holes, accordingly improving the injection efficiency of holes. The related physical mechanism is similar to the case of the electrons at the conduction band in sample S_up_. As a result, due to the joint impact of the decreased hole potential barrier height and the enhanced quantum tunneling process for holes, the hole injection process is promoted, and thereby the hole concentration in the active region is increased, as illustrated in Figure 4b,d. Overall, the CLQB layer in sample S_down_ can effectively suppress electron overflow and facilitate hole injection, thereby enhancing carrier concentration in the MQW layers and promoting the radiative recombination process in the active region.

**Figure 6:**
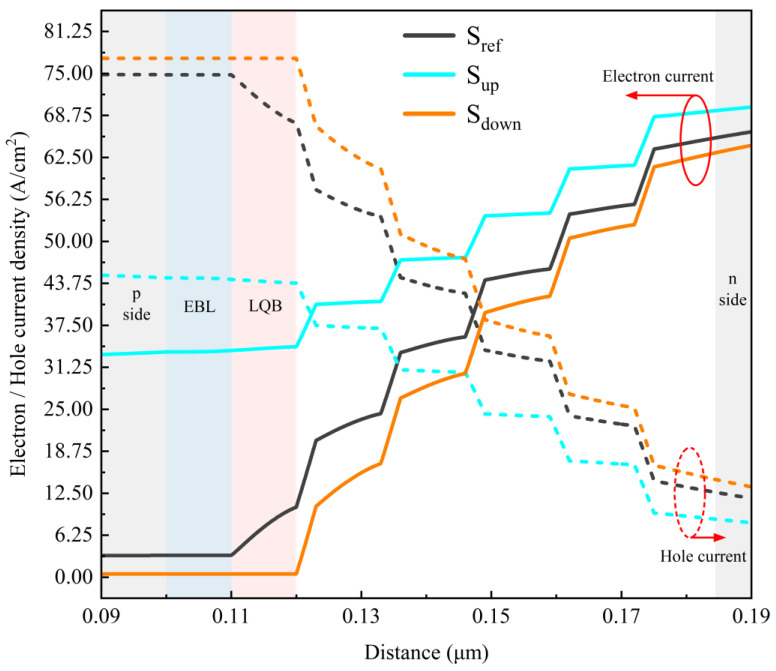
Electron (solid lines) and hole (dot lines) current densities in active region; LQB, EBL, and CLQB layers for all samples.

To further validate the aforementioned discussion, the distributions of electron and hole current density at an injection current of 500 mA for all of the samples are depicted in Figure 6. It can be seen that sample S_down_ exhibits the smallest electron leakage current density of about 1 A/cm^2^, while sample S_up_ has the largest electron leakage current density of about 34 A/cm^2^. Thus, compared to sample S_ref_ with the traditional EBL/LQB structure, it is obvious that the electron blocking capability of the CLQB layer is enhanced for sample S_down_, while it is weak for sample S_up_. Additionally, it is noteworthy that for sample S_ref_, there is an obvious variation in the electron and hole current densities in the LQB region, which shows almost no change in other two samples with CLQB structures, indicating the presence of accumulation and recombination loss of electrons and holes in the LQB layer in sample S_ref_, as discussed in Figure 3, Figure 4 and Figure 5. At last, it should be pointed out that it seems a little strange that the hole injection current is the highest for sample S_down_ while it is the lowest for sample S_up_ in Figure 6, although the effective hole barrier heights of the CLQB layers of both samples are similar in Figure 5. In fact, one should keep in mind that the hole injection current depends not only on the effective barrier height at the conduction band, but also on the leakage current of electrons. If there are plenty of electrons leaking into the p-type region, a large number of leaking electrons can recombine with the holes injected from the p-type region, eventually resulting in a significant decrease in holes injected into the MQW region. Therefore, since the electron leakage in sample S_up_ is remarkable, the number of holes injected into the MQW active region is diminished.

## 4. Conclusions

In summary, to suppress the electron leakage and promote hole injection of AlGaN-based DUV LEDs, the traditional EBL and LQB structures are replaced with the Al content-varied AlGaN CLQB layer, where the average Al content in the CLQB layer of samples S_up_ and S_down_ is identical to that in the traditional EBL/LQB structure in sample S_ref_. The effects of the CLQBs with different distributions of Al content on the carrier transport characteristics of AlGaN-based DUV LEDs were carefully numerically studied. This work shows that, in sample S_down_, a CLQB with the Al content decreased from 70% to 60% towards the growth direction can effectively suppress the electron leakage current and facilitate the hole injection process simultaneously. Additionally, it can also prevent the carrier accumulation and recombination loss at the EBL/LQB hetero-interface in the traditional structures, which further increases the carrier concentration and leads to an enhanced radiative recombination process in the MQW active regions. Consequently, the luminescence efficiency of sample S_down_ is improved significantly. It should be emphasized that although the improvement of the peak IQE is less significant for sample S_down_, its efficiency droop is remarkably relieved by the optimized CLQB structure at high injection currents, making our research meaningful and valuable. Finally, for the actual LEDs, the fabrication process of an Al-content-varied CLQB is very challenging. Therefore, a step-shaped CLQB structure may be adopted to achieve a better balance between fabrication difficulty and performance improvement in future work.

## Data Availability

The original contributions presented in the study are included in the article, further inquiries can be directed to the corresponding authors.
